# Comparison of the triglyceride/high-density lipoprotein ratio, lipid profile, and glycated hemoglobin and their association with coronary artery disease in the older adult Saudi population: a case-control study

**DOI:** 10.3389/fcvm.2026.1835214

**Published:** 2026-06-19

**Authors:** Thamir Al-khlaiwi, Ayman Alsaleh, Hessah Alshammari, Sara Abou Al-Saud, Manan Alhakbany, Abdulmalik Alqahtani, Aliah Alshanwani, Sarah Mazi, Muhammad Iqbal

**Affiliations:** 1 Department of Physiology, College of Medicine, King Saud University, Riyadh, Saudi Arabia; 2 Department of Cardiac Sciences, King Fahad Cardiac Center, College of Medicine, King Saud University Medical City, King Saud University, Riyadh, Saudi Arabia; 3 Department of Cardiac Sciences, College of Medicine, King Saud University, Riyadh, Saudi Arabia

**Keywords:** coronary artery disease, predictor, Saudi population, sensitivity, triglyceride/high-density lipoprotein ratio

## Abstract

**Background and objective:**

There is a lack of information regarding ethnic variability in the association between the triglyceride/high-density lipoprotein (TG/HDL) ratio and coronary artery disease (CAD), particularly in the Saudi population. This study aimed to evaluate the association, sensitivity, and specificity of TG/HDL ratio in relation to CAD in the Saudi population.

**Methods:**

This study is a retrospective, analytical case–control study. Data were obtained from patients' electronic medical records at King Saud University Medical City (KSUMC) between 2015 and 2023. The vessel scores and Gensini score were used to assess the extent of vessel involvement and the severity of coronary occlusion. Participants were divided into two groups: (1) a healthy control group with no CAD and aged over 50 years, and (2) patients with CAD aged over 50 years. Patients with CAD underwent selective coronary angiography according to the hospital's routine procedures. Biochemical markers were measured at the time of coronary angiography.

**Results:**

Multivariable logistic regression model showed that TG/HDL ratio was significantly associated with CAD (OR = 1.87, 95% CI [1.35, 2.58], *p* < 0.001). HbA1c demonstrated the strongest predictive ability, yielding an excellent area under the ROC curve (AUC = 0.837, 95% CI [0.807, 0.866], *p* < 0.001). High-density lipoprotein (HDL) levels also showed good discriminative performance (AUC = 0.716, 95% CI [0.682, 0.750], *p* < .001). The TG/HDL ratio demonstrated modest but statistically significant predictive ability (AUC = 0.630, 95% CI [0.592, 0.667], *p* < 0.001), whereas serum triglycerides (TG) levels alone showed poor-to-fair discrimination (AUC = 0.556, 95% CI [0.516, 0.596], *p* = 0.006). Regarding sensitivity/specificity values, the best cut-off value for the TG/HDL ratio was 1.41, sensitivity of 59% and specificity of 64%.

**Conclusion:**

The TG/HDL ratio was significantly associated with the occurrence of CAD in Saudi population aged over 50 years. However, it was less sensitive and specific than HDL and HbA1c as biomarkers for detecting CAD. Overall, these findings indicate that HbA1c is the most powerful biomarker for predicting CAD among adults aged ≥ 50 years.

## 1 Introduction

Cardiovascular diseases (CVD) are major contributors to global mortality and represent a significant health challenge worldwide (1). Saudi Arabia has one of the highest CVD-related mortality rates among Gulf Cooperation Council (GCC) countries, estimated to range between 329.5 and 379.6 per 100,000 populations. These rates are higher than those reported in high-income North America (99.4-and 191.6 per 100,000) and Western Europe (80.2–199.9 per 100,000) [1]. A comprehensive study conducted in Saudi Arabia by the General Authority for Statistics estimated the prevalence of CVD to be 1.9% prevalence in males and 1.4% in females across all regions (2).

One of the main factors influencing CVD, particularly CAD, is dyslipidaemia, which is defined as abnormal levels of lipids in the blood. It is a major risk factor contributing to both the morbidity and mortality associated with CVD. Dyslipidaemia is characterised by elevated levels of TC, TG, LDL, along with alterations in HDL (3, 4). A study conducted across all regions of Saudi Arabia reported that one-fourth of the adolescent population (males: 33.3%, females: 17.9%) had dyslipidaemia (5).

Serum TG, TC, LDL, HDL, along with TC/HDL and LDL/HDL ratios, serve as independent predictors of cardiovascular disease risk. Each of these markers plays a significant role in both incidence and prognosis of CAD. For example, lowering LDL levels with medication such as statin can reduce the risk of atherosclerotic cardiovascular disease (ASCVD) (6). Conversely, low HDL and high TG levels, particularly when the fasting TG levels are ≥150 mg/dL, are associated with an increased the risk of CAD (7).

Therefore, early assessment of dyslipidaemia in patients is crucial for the management and prevention of atherosclerosis. One of the promising markers highlighted in the literature is the TG/HDL ratio, an atherogenic index and a reliable marker for predicting the risk of CAD, insulin resistance, and metabolic syndrome (8). Across various countries and studies, this ratio has demonstrated good sensitivity and specificity in predicting the extent of coronary atherosclerosis.

Evidence from the literature suggests a strong association between TG/HDL ratio and the risk of CAD (9, 10). The TG/HDL ratio, along with other traditional risk factors such as hypertension, diabetes, smoking and dyslipidaemia, can help identify individuals at high risk of developing CAD, enabling timely management and intervention and thereby reducing the likelihood of adverse cardiovascular complications.

Due to the lack of previous studies and the ethnic diversity, we aimed to evaluate the association, sensitivity, and specificity of TG/HDL ratio with CAD in the Saudi population. This will help in the early screening, assessment, and prognosis of this serious health condition.

## 2 Method

**Figure 1:**
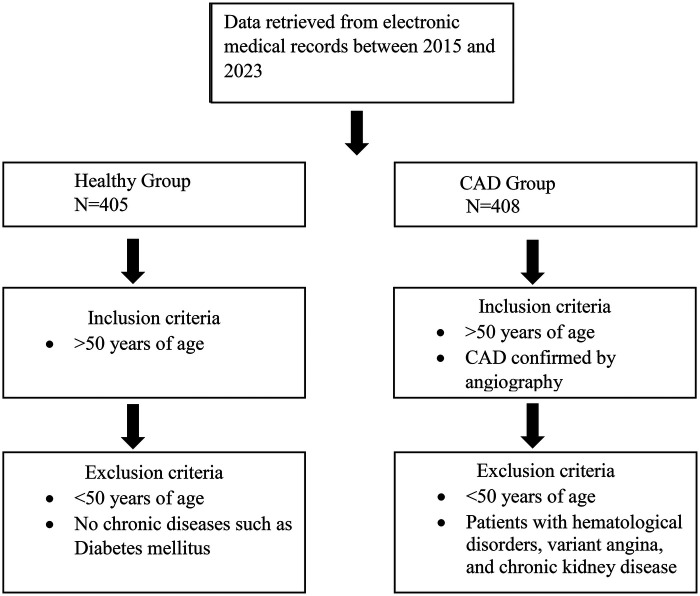
Flow diagram for patient selection method.

This retrospective analytical case-control study was conducted in the Department of Physiology, College of Medicine, King Saud University, Riyadh, Saudi Arabia. Data were retrieved from electronic medical records at King Saud University Medical City (KSUMC) between 2015 and 2023, including sociodemographic data (age, gender, BMI, smoking status, etc.), lipid profile (LDL, HDL, total cholesterol, triglycerides), HbA1c, creatinine levels, blood pressure, and detailed medical history of chronic diseases. The study population was divided into two groups: (1) The healthy group (non-CAD), consisting of individuals with no evidence of CAD and aged over 50 years, as evaluated by a cardiologist. Data for this group were collected retrospectively from the electronic blood bank system. Inclusion criteria for healthy subjects were selected using a convenience sampling technique to determine the cut-off values for the TG/HDL ratio while minimizing the influence of chronic diseases such as diabetes, which was an exclusion criterion for the healthy group. (2) The CAD group, consisting of patients aged over 50 years with CAD confirmed by angiography. Inclusion criteria for the CAD group included males and females older than 50 years with angiographically confirmed CAD. The exclusion criteria included patients with hematological disorders, variant angina, and chronic kidney disease (Figure 1).

CAD patients underwent coronary angiography via the right femoral artery approach, following the hospital's standard protocol. Imaging was assessed using right and left oblique views in cranial and caudal positions. Coronary artery involvement and severity of occlusion were evaluated using vessel and Gensini scores. The vessel score assessed significant stenosis (>50%) in the major coronary arteries: left anterior descending (LAD), left circumflex (LCX), and right coronary artery (RCA), classifying patients as having one-, two-, or three-vessel disease. The Gensini score quantified the location, extent, and degree of arterial occlusion, with a score of zero indicating no occlusion. Occlusion in at least one of the major coronary arteries (left main artery (LM), LAD, LCX, or RCA) qualified a patient for inclusion in the CAD group, which included both acute coronary syndrome cases and patients undergoing diagnostic evaluation for suspected chronic coronary syndrome. Biochemical markers were obtained at the time of coronary angiography.

The study was approved by the Institutional Review Board (IRB) of the College of Medicine, King Saud University (No. E-22-6747) in accordance with the Declaration of Helsinki. Participants' privacy and confidentiality were strictly maintained.

### 2.1 Statistical analysis

Continuous variables were described using mean and standard deviation, while categorical variables were summarized as frequencies and percentages. For questions allowing multiple responses, multiple response dichotomies analysis was applied. Associations between categorical variables were assessed using the chi-squared test of independence. Differences in means for metric variables between dichotomous groups were evaluated using the independent samples *t*-test. The diagnostic performance of the TG/HDL ratio for CAD was assessed using the area under the receiver operating characteristic curve (AUC-ROC). The AUC-ROC, together with Youden Index analysis, was used to evaluate the diagnostic ability of the TG/HDL ratio and other biomarkers in predicting CAD (10, 14). Multivariable binary logistic regression (MBLR) was employed to identify significant predictors of CAD. Associations between independent predictor variables and the outcome were expressed as adjusted odds ratios with 95% confidence intervals. Predictor variables were selected based on a literature review and bivariate analyses; relevant predictors were entered sequentially into iterative models to achieve parsimony and optimize diagnostic accuracy. All statistical analyses were performed using IBM SPSS Version 29, with a significance level set at *α* = 0.05.

## 3 Results

### 3.1 Sociodemographic characteristics of total sample size, healthy, and CAD group (*n* = 812)

The study included 812 adults residing in Saudi Arabia, aged 51 years and older, comprising both individuals with a prior diagnosis of CAD and those without known CAD. Table 1 presents bivariate comparisons of sociodemographic and clinical characteristics between participants without CAD and those diagnosed with CAD (*N* = 812). Regarding sex distribution, 53.3% of females were in the non-CAD group and 17.4% in the CAD group, whereas 46.7% of males were non-CAD and 82.6% were CAD. A chi-square test showed a significant association between sex and CAD status, *χ*²(1) = 114.41, *p* < 0.001.

**Table 1:** Descriptive analysis of sample's sociodemographic characteristics. *N* = 812.

Variable	Total sample size	Healthy group (*n* = 405)	CAD group (*n* = 407)	test statistic	*p*-value
Sex					
Female	287 (35.3)	216 (53.3)	71 (17.4)	χ²(1) = 114.41	<0.001
Male	525 (64.7)	189 (46.7)	336 (82.6)		
Age (years), mean (SD)	61 (7.51)	62.94 (7.75)	61.97 (8.32)	t(810) = 1.73	0.085
Age group					
51–60 years	395 (48.6)	182 (44.9)	161 (40.5)	χ²(3) = 2.89	0.239
61–70 years	304 (37.4)	174 (43)	182 (43.7)		
71–80 years	113 (13.9)	49 (12.1)	64 (15.7)		
Body weight (Kg), mean (SD)	75.14 (12.63)	71.66 (9.71)	78.59 (14.17)	t(718.57) = 8.14	<0.001
Body Height (cm), mean (SD)	163.35 (9.1)	161.77 (101)	164.92 (7.80)	t(760.03) = 4.99	<0.001
Body Mass Index (BMI) score, mean (SD)	28.10 (3.78)	27.36 (2.54)	28.79 (4.60)	t(632.95) = 5.51	<0.001
Body Mass Index (BMI) score level					
Underweight	10 (1.2)	2 (0.5)	8 (2)		
Normal	146 (18)	72 (17.8)	74 (18.2)	χ²(4) = 77.53	<0.001
Over-weight	443 (54.6)	268 (66.2)	175 (43)		
Obesity class I	170 (20.9)	63 (15.6)	107 (26.3)		
Obesity class II	43 (5.3)	0	43 (10.6)		
Nationality					
Saudi	645 (79.4)	383 (94.6)	262 (64.4)	χ²(1) = 113.30	<0.001
Non-Saudi	167 (20.6)	22 (5.4)	145 (35.6)		
Medical History of Diabetes	242 (29.8)	0	242 (59.2)	χ²(1) = 343.1	<0.001
Medical History of Hypertension	220 (27.1)	0	219 (53.8)	χ²(1) = 294.86	<0.001
Smoking					
None/never smoker	651 (80.2)	392 (96.8)	259 (63.6)	χ²(2) = 140.4	<0.001
Ex-smoker	17 (2.1)	1 (0.2)	16 (3.9)		
Currently smoker	144 (17.7)	12 (3)	132 (32.4)		

Body mass index (BMI) also differed significantly, with a mean of 27.36 (SD = 2.54) among non-CAD participants and 28.79 (SD = 4.60) among CAD participants, t(632.95) = 5.51, *p* < 0.001.

BMI classification varied across groups, *χ*²(4) = 77.53, *p* < 0.001. In the non-CAD group, 0.5% were underweight, 17.8% normal weight, 66.2% overweight, and 15.6% obese class I, with no participants in obese class II. In the CAD group, 2% were underweight, 18.2% normal weight, 43% overweight, 26.3% obese class I, and 10.6% obese class II.

History of diabetes differed between groups, *χ*²(1) = 343.1, *p* < 0.001. All non-CAD participants (100%) reported no diabetes, whereas 59.2% of CAD patients reported diabetes. Similarly, hypertension history differed significantly, *χ*²(1) = 294.86, *p* < 0.001, with 100% of non-CAD participants reporting no hypertension vs. 53.8% of CAD participants reported hypertension.

Smoking status also showed a significant association with CAD status, *χ*²(2) = 140.4, *p* < 0.001.

In the non-CAD group, 96.8% were never-smokers, 0.2% were ex-smokers, and 3% were current smokers. In the CAD group, 63.6% were never-smokers, 3.9% were ex-smokers, and 32.4% were current smokers.

### 3.2 Clinical parameters of total sample size, healthy group, and CAD group (*N* = 812)

Table 2 compares laboratory measurements between participants without CAD and those diagnosed with CAD (*N* = 812). Systolic blood pressure did not differ significantly between groups, with mean values of 130.31 mmHg (SD = 13.67) in non-CAD participants and 131.47 mmHg (SD = 26.56) in CAD participants, t(607.6) = 0.784, *p* = 0.433. Diastolic blood pressure was higher in the CAD group, 76.50 mmHg (SD = 15.97), compared to 73.94 mmHg (SD = 8.61) in the non-CAD group, t(624.50) = 2.84, *p* = 0.005. Mean arterial pressure also differed between groups—92.73 mmHg (SD = 8.44) in the non-CAD group and 94.82 mmHg (SD = 17.73) in the CAD group, t(581.7) = 2.15, *p* = 0.032.

**Table 2:** Descriptive analysis of clinical characteristics of sample size (*N* = 812).

Variable	Total sample size	Healthy group	CAD group	test statistic	*p*-value
Systolic Blood Pressure (mmHg), mean (SD)	130.89 (21.14)	130.31 (13.67)	131.47 (26.56)	t(607.6) = 0.784	0.433
Diastolic Blood Pressure (mmHg), mean (SD)	75.22 (12.89)	73.94 (8.61)	76.50 (15.97)	t(624.50) = 2.84	0.005
Mean Arterial Blood (MAP) Pressure (mmHg)	93.78 (13.93)	92.73 (8.44)	94.82 (17.73)	t(581.7) = 2.15	0.032
Serum Glycate (HbA1c) haemoglobin (%) level, mean (SD)	6.54 (1.58)	5.74 (0.41)	7.34 (1.87)	t(443.5) = 16.6	<0.001
Serum HbA1c level					
Low/normal <6.5%	575 (70.8)	405 (100)	170 (41.8)	χ² (1) = 333.04	<0.001
High ≥6.5%	237 (29.2)	0	237 (58.2)		
Serum Total Cholesterol level (mmol/L), mean (SD)	4.46 (1.16)	4.40 (0.914)	4.52 (1.36)	t(710.6) = 1.53	0.127
Serum total cholesterol level					
Normal <5.2 mmol/L	621 (76.5)	349 (86.2)	272 (66.8)	χ² (1) = 42.22	<0.001
High ≥5.2 mmol/L	191 (23.5)	56 (13.8)	135 (33.2)		
Serum Low Density Lipoprotein (LDL) level (mmol/L), mean (SD)	2.48 (1.12)	2.46 (0.93)	2.51 (1.29)	t(738.8) = 0.558	0.576
Serum LDL level					
LDL≤2.85 mmol/L	540 (66.5)	289 (71.4)	251 (61.7)	χ² (1) = 8.55	0.003
LDL>2.85 mmol/L	272 (33.5)	116 (28.6)	156 (38.3)		
Serum Triglyceride (TG) level (mmol/L), mean (SD)	1.54 (0.91)	1.33 (0.50)	1.75 (1.53)	t(543.14) = 6.84	<0.001
Serum TG level					
TG ≤1.7 mmol/L	561 (69.1)	321 (79.3)	240 (59)	χ^2^ (1) = 39.14	<0.001
TG >1.7 mmol/L	251 (30.9)	84 (20.7)	167 (41)		
Serum High Density Lipoprotein (HDL) level (mmol/L), mean (SD)	1.18 (0.4)	1.31 (0.36)	1.05 (0.40)	t(810) = 9.37	<0.001
Serum HDL level					
Normal	418 (51.5)	248 (61.2)	170 (41.8)	χ² (1) = 33.7	<0.001
Low	394 (48.5)	157 (38.8)	237 (58.2)		
Triglyceride/ High Density Lipoprotein Ratio (TG/HDL), mean (SD)	1.56 (1.29)	1.12 (0.53)	1.99 (1.64)	t(490.15) = 10.2	<0.001

HbA1c levels showed marked group differences. The mean HbA1c was 5.74% (SD = 0.41) among participants without CAD and 7.34% (SD = 1.87) among those with CAD, t(443.5) = 16.6, *p* < 0.001. When categorized, 100% of non-CAD participants had HbA1c < 6.5%, while 41.8% of CAD participants were in the low/normal category and 58.2% in the high category, *χ*²(1) = 333.04, *p* < 0.001.

The mean total cholesterol level was 4.40 mmol/L (SD = 0.914) in the non-CAD group and 4.52 mmol/L (SD = 1.36) in the CAD group, with no significant difference, t(710.6) = 1.53, *p* = 0.127. However, categorical cholesterol levels differed significantly: 86.2% of non-CAD participants vs. 66.8% of CAD participants were below 5.2 mmol/L, *χ*²(1) = 42.22, *p* < 0.001.

Mean LDL level did not differ significantly—2.46 mmol/L (SD = 0.93) in the non-CAD group and 2.51 mmol/L (SD = 1.29) in the CAD group, t(738.8) = 0.558, *p* = 0.576. Categorical LDL levels showed a significant difference, with 71.4% of non-CAD participants and 61.7% of CAD participants having LDL ≤2.85 mmol/L, *χ*²(1) = 8.55, *p* = 0.003.

Mean TG levels were 1.33 mmol/L (SD = 0.50) in participants without CAD and 1.75 mmol/L (SD = 1.53) in those with CAD, t(543.14) = 6.84, *p* < 0.001. Categorically, 79.3% of non-CAD participants had TG ≤ 1.7 mmol/L compared with 59% of CAD participants, *χ*²(1) = 39.14, *p* < 0.001.

Serum HDL levels were significantly lower in the CAD group: 1.05 mmol/L (SD = 0.40) compared with 1.31 mmol/L (SD = 0.36) in the non-CAD group, t(810) = 9.37, *p* < 0.001. Sex-standardized HDL values also showed significant group differences: 61.2% of non-CAD participants vs. 41.8% of CAD participants had HDL in the normal-for-sex range, *χ*²(1) = 33.7, *p* < 0.001.

Finally, the TG/HDL ratio differed significantly between groups. The non-CAD group had a mean TG/HDL ratio of 1.12 (SD = 0.53), whereas the CAD group had a mean of 1.99 (SD = 1.64), t(490.15) = 10.2, *p* < 0.001.

### 3.3 Assessment of affected coronary vessels and related risk scores (*n* = 407)

Regarding the number of major vessels involved, 12.7% had one affected vessel, 17.7% had two affected vessels, and 69.6% had three affected major vessels. The LAD was the most commonly affected vessel, noted in 382 patients (97.1%).

**Figure 2:**
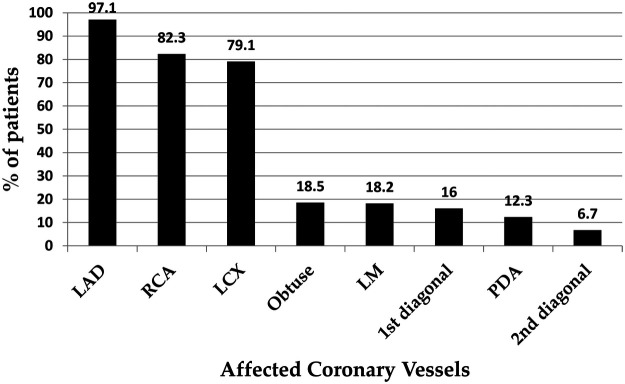
The distribution of affected coronary vessels among CAD patients, *n* = 407.

The RCA was affected in 334 patients (82.3%), and the LCX in 321 patients (79.1%). Additional vessel involvement included the first diagonal branch in 65 patients (16%), the second diagonal branch in 27 patients (6.7%), the left main (LM) artery in 75 patients (18.2%), the posterior descending artery (PDA) in 50 patients (12.3%), and the obtuse marginal branch in 75 patients (18.5%) (Figure 2). Coronary Risk scoring indices are also reported, with a median Gensini score of 62 (IQR = 49), and a median Coronary Vessel Score of 3 (IQR = 1) among this CAD-diagnosed subgroup.

### 3.4 Bivariate analysis of gender-based comparison of affected vessels and risk score (*n* = 407)

Table 3 presents gender-based comparisons of coronary vessel involvement and risk scores among 407 patients diagnosed with CAD. According to the Mann–Whitney U non-parametric test, female elderly CAD patients had a significantly lower serum TG/HDL ratio (median score = 1.15) compared to male patients (median score=1.44), *p* = 0.007. Median Gensini scores were 56 (IQR = 46) for females and 64 (IQR = 50.5) for males. The Mann–Whitney test indicated no statistically significant difference between genders (*Z* = 1.46, *p* = 0.144). Similarly, the median vessel score was 0.830 (IQR = 1) for females and 0.71 (IQR = 1) for males, with no significant difference detected (*Z* = 0.933, *p* = 0.351). Regarding vessel involvement, LAD was affected in 91.5% of females and 94.4% of males, with no significant association (*χ*²(1) = 0.392, *p* = 0.531). The first diagonal branch was affected in 15.7% of females and 16.3% of males (*χ*²(1) = 0.016, *p* = 0.900), and the second diagonal branch in 4.3% of females and 7.4% of males (*χ*²(1) = 0.466, *p* = 0.459). Left main artery involvement occurred in 20% of females and 18.1% of males (*χ*²(1) = 0.140, *p* = 0.709). The RCA was affected in 78.6% of females and 83.1% of males (*χ*²(1) = 0.811, *p* = 0.368). PDA involvement was recorded in 8.6% of females and 13.4% of males (*χ*²(1) = 1.23, *p* = 0.268). The LCX was affected in 77.1% of females and 79.5% of males (*χ*²(1) = 0.199, *p* = 0.655), while obtuse marginal branch involvement occurred in 21.4% of females and 17.8% of males (*χ*²(1) = 0.507, *p* = 0.477).

**Table 3:** Descriptive bivariate analysis of gender differences on coronary affected vessels and risk scores among CAD diagnosed people, *n* = 407.

Variable	Female	Male	test statistic	*p*-value
TG/HDL ratio, median (IQR)	1.15 (1.45)	1.44 (1.81)	*Z* = 2.72, DF = 408	0.007
Gensini score, median (IQR)	56 (46)	64 (50.5)	*Z* = 1.46, df = 408	0.144
Vessel score, median (IQR)	0.830 (1)	0.71 (1)	*Z* = 0.933, df = 408	0.351
Affected coronary vessels				
LAD	65 (91.5)	318 (94.4)	χ² (1) = 0.392	0.531
1st diagonal	11 (15.7)	55 (16.3)	χ² (1) = 0.016	0.9
2nd diagonal	3 (4.3)	25 (7.4)	χ² (1) = 0.466	0.459
LM	14 (20)	61 (18.1)	χ² (1) = 0.14	0.709
RCA	55 (78.6)	280 (83.1)	χ² (1) = 0.811	0.368
PDA	6 (8.6)	45 (13.4)	χ² (1) = 1.23	0.268
LCX	54 (77.1)	268 (79.5)	χ² (1) = 0.199	0.655
Obtuse	15 (21.4)	60 (17.8)	χ² (1) = 0.507	0.477

### 3.5 Multivariable binary logistic regression analysis of CAD

Table 4 presents the multivariable binary logistic regression analysis examining the independent predictors of a diagnosis of CAD (No/Yes). The model included gender, age, nationality, body mass index (BMI), mean arterial blood pressure, TG/HDL ratio, and smoking status as covariates. Adjusted odds ratios (AORs) with 95% confidence intervals (CIs) were estimated.‏

**Table 4:** Multivariable logistic binary regression analysis of CAD patients.

	Multivariable adjusted Odds Ratio	95.0% CI for OR		*p*-value
		Lower Bound	Upper Bound	
Gender = Male Vs Female	2.920	1.965	4.339	<0.001
Mean Age (years)	1.019	0.997	1.041	0.088
Nationality = Saudi vs. Non-Saudi	10.953	6.263	19.157	<0.001
Mean Body Mass Index (BMI) score	1.208	1.143	1.278	<0.001
Mean Arterial Blood Pressure (mmHg)	1.006	0.992	1.020	0.382
Mean TG/HDL Ratio	1.903	1.549	2.338	<0.001
High Density Lipoprotein (HDL) level	1.364	0.861	2.160	0.185
Glycated (HbA1C) hemoglobin index	9.54	5.542	21.121	<.001
Smoking: smoker Vs None smoker	4.897	3.362	7.133	<0.001
Constant	0.000			<0.001

Dependent outcome: Diagnosis with Coronary artery disease, CAD, (No/Yes).

After controlling for all other variables in the model, male gender was significantly associated with higher odds of CAD compared with female (AOR = 2.92, 95% CI [1.97, 4.34], *p* < 0.001). This indicates that males had nearly three times the odds of being diagnosed with CAD relative to females.‏ Age was not a statistically significant predictor of CAD in the adjusted model (AOR = 1.02, 95% CI [1.00, 1.04], *p* = 0.088), although the direction of effect suggested a small positive association per one-year increase in age. Nationality demonstrated a strong and statistically significant association with CAD. Saudi participants had markedly higher odds of CAD compared with non-Saudi participants (AOR = 10.95, 95% CI [6.26, 19.16], *p* < 0.001), indicating nearly eleven-fold increased odds after adjustment for other covariates. Body mass index was also a significant independent predictor. For each one-unit increase in mean BMI, the odds of CAD increased by approximately 21% (AOR = 1.21, 95% CI [1.14, 1.28], *p* < 0.001), suggesting a clinically meaningful association between higher BMI and coronary heart disease. Mean arterial blood pressure was not significantly associated with CAD in the multivariable model (AOR = 1.01, 95% CI [0.99, 1.02], *p* = 0.382).

The TG/HDL ratio was a strong independent predictor of CAD. Each one-unit increase in the TG/HDL ratio was associated with nearly doubled odds of CAD (AOR = 1.90, 95% CI [1.55, 2.34], *p* < 0.001), highlighting its substantial contribution to coronary disease risk.

Smoking status was also significantly associated with CAD. Individuals who were smokers had approximately 4.90 times higher odds of CAD compared with non-smokers (AOR = 4.90, 95% CI [3.36, 7.13], *p* < 0.001).

### 3.6 AUC-ROC analysis of the TG/HDL ratio for CAD

**Figure 3:**
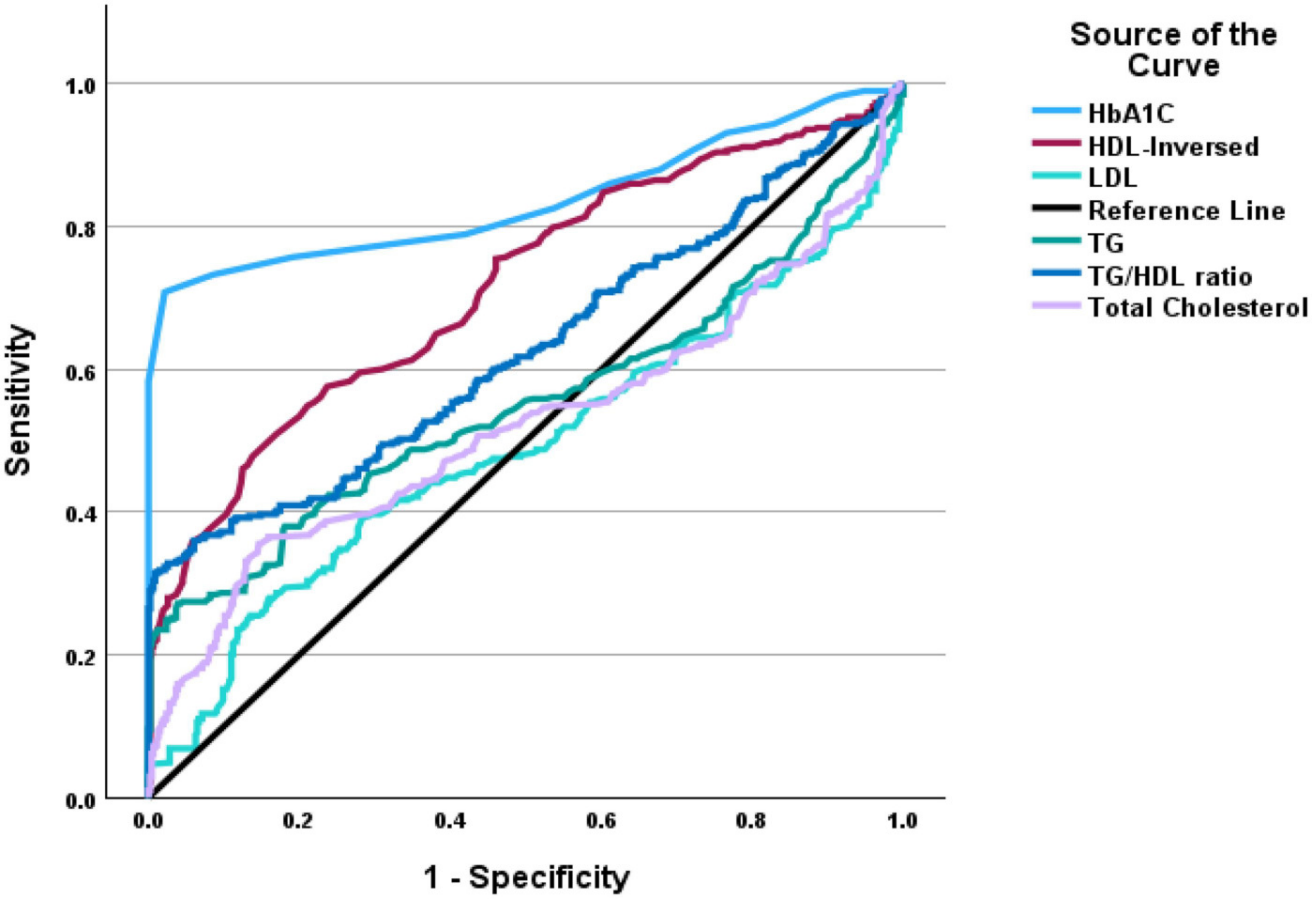
ROC curve analysis of biomarkers predicting for CAD.

Receiver operating characteristic (ROC) curve analysis was performed to evaluate the discriminatory performance of several blood biomarkers for predicting CAD among adults aged ≥51 years. The results are presented Table 5 and Figure 3 (AUC-ROC). Among the evaluated markers, HbA1c showed the strongest predictive ability, with an excellent area under the ROC curve (AUC = 0.837, 95% CI [0.807, 0.866], *p* < 0.001), indicating high accuracy in distinguishing individuals with and without CAD in this age group. HDL levels also demonstrated good discriminative performance (AUC = 0.716, 95% CI [0.682, 0.750], *p* < 0.001), supporting its role as a protective lipid marker in older adults.

**Table 5:** Area under the ROC curve analysis of various blood biomarkers predicting coronary artery disease among adults aged ≥51 years.

Test Result Variable(s)	Area Under ROC	Lower Bound	Upper Bound	*p*-value
TG/HDL Ratio	0.630	0.592	0.667	<0.001
Serum Triglyceride (TG)	0.556	0.516	0.596	0.006
Serum High density Lipoprotein (HDL) score	0.716	0.682	0.750	<0.001
Serum glycated haemoglobin (HbA1c)	0.837	0.807	0.866	<0.001
Serum total Cholesterol	0.523	0.483	0.563	0.261
Serum Low density Lipoprotein (LDL) score	0.493	0.453	0.533	0.733

The TG/HDL ratio exhibited modest but statistically significant predictive ability (AUC = 0.630, 95% CI [0.592, 0.667], *p* < 0.001), whereas serum TG levels alone showed poor-to-fair discrimination (AUC = 0.556, 95% CI [0.516, 0.596], *p* = 0.006). In contrast, TC did not significantly discriminate CAD status (AUC = 0.523, 95% CI [0.483, 0.563], *p* = 0.261), and LDL cholesterol performed no better than chance (AUC = 0.493, 95% CI [0.453, 0.533], *p* = 0.733).

### 3.7 AUC-ROC analysis of the TG/HDL ratio as a diagnostic discriminator for CAD

The ROC analysis evaluating the TG/HDL ratio as a diagnostic discriminator of CAD in older adults yielded a low AUC of 0.630 (95% CI: 0.601–0.678), indicating only modest accuracy and a substantial overlap between CAD and non-CAD distributions. Examination of the Youden Index further supported this limited discriminatory performance. Although several cut-off points produced statistically acceptable sensitivity–specificity combinations, the highest Youden values clustered around low to moderate TG/HDL thresholds, none surpassing levels typically associated with strong diagnostic tests. In particular, even the best-performing thresholds produced only modest gains in sensitivity or specificity, confirming that no single TG/HDL cut-off meaningfully separates CAD from non-CAD groups in this elderly population (Table 6).

**Table 6:** Best-performing TG/HDL cut-offs according to youden index metrics.

Number of cut off	TG/HDL Cut-off	Sensitivity	Specificity	Youden Index
1	1.41	0.59	0.64	0.23
2	1.38	0.6	0.63	0.22
3	1.35	0.61	0.61	0.22
4	1.48	0.56	0.66	0.22
5	1.52	0.54	0.67	0.21

## 4 Discussion

Our findings indicate that, among the Saudi population aged over 50 years, the TG/HDL ratio and total cholesterol were significantly associated with the occurrence of CAD and were more prevalent in males than in females. In contrast, HbA1c and HDL levels demonstrated higher sensitivity and specificity than TG/HDL ratio and total cholesterol for predicting CAD in this population.

These findings are consistent with previous studies identifying the TG/HDL as an indicator of CAD risk. Yang et al. (11) reported that an elevated TG/HDL ratio were associated with an increased risk of cardiovascular events in patients with diabetes and stable cardiovascular disease (11). Similarly, Wang et al. (12) identified the TG/HDL ratio as an independent risk factor and a useful predictor of coronary artery calcification (12). A high TG/HDL ratio is associated with high-risk plaques identified by coronary computed tomography angiography, suggesting a potential biomarker for assessing CAD risk (13). Associations between the TG/HDL ratio and other cardiovascular risk factors, such as myocardial infarction, carotid intima–media thickness, arterial stiffness, and insulin resistance, have also been reported (14). We recently observed a significant correlation between the TG/HDL ratio and coronary artery calcium scores in patients at risk of coronary artery disease within the Saudi population (15).

However, evidence regarding the independent predictive value of the TG/HDL ratio remains inconsistent. A J- or U-shaped relationship between HDL levels and cardiovascular mortality has been described (16, 17), suggesting that both low and high HDL concentrations may be associated with increased cardiovascular risk (18). Conversely, other studies have reported no association between high HDL levels and mortality (19). There are also reports describing no significant association between the TG/HDL ratio and major cardiovascular events (20).

Luca et al. (21) further demonstrated that the TG/HDL ratio was not associated with the prevalence or extent of CAD, whereas HDL levels were significantly associated with disease burden (21). Collectively, these findings suggest that, beyond the TG/HDL ratio, additional parameters, including HDL, HbA1c, and smoking status, should be considered when predicting CAD. Furthermore, HDL functionality, physiological maturation, and genetic factors may influence its role in cardiovascular disease.

In our study, smoking status and HbA1c levels were among the most sensitive risk factors for predicting CAD in our patients. HbA1c reflects average blood glucose levels over approximately 2–3 months and has been correlated with CAD in both diabetic and non-diabetic individuals and is associated with endothelial dysfunction, inflammation, oxidative stress and atherosclerosis (22–27). In the present analysis, HbA1c demonstrated the strongest predictive ability for CAD, with an excellent area under the ROC curve (AUC = 0.837), indicating excellent discriminative performance. In contrast, HDL showed good predictive ability (AUC = 0.716), whereas the TG/HDL ratio demonstrated modest but statistically significant discrimination (AUC = 0.630). Serum triglycerides alone showed poor discriminatory performance (AUC = 0.556). These findings indicate that chronic hyperglycemia may be more strongly associated with CAD than isolated lipid abnormalities.

The incidence and prevalence of diabetes mellitus are increasing across the Arab region and appear to exceed global rates. In the Middle East and North Africa (MENA) region, one in six adults aged 20–79 years is affected by type 2 diabetes mellitus (T2DM). Saudi Arabia ranks seventh globally in the prevalence of diabetes mellitus, and the prevalence of T2DM among adults is estimated at 28% (28). Furthermore, projections suggest that the prevalence of T2DM in Saudi Arabia may rise to 40.37% by 2025 and 45.36% by 2030. The IDF also estimates that approximately one quarter of Saudi adults could be living with diabetes by 2045 (29).

Multiple factors contribute to the increasing prevalence of T2DM in this region, particularly in Saudi Arabia. Rapid urbanization and economic development have led to a rise in the standard of living, adoption of unhealthy diets high in sugar, reduced physical activity, and a high prevalence of overweight and obesity, all of which are established risk factors for T2DM in the Saudi Arabian population (30). An earlier study reported that approximately one quarter of participants had inadequate glycemic control (HbA1c ≥7%) (31). Genetic factors also play a significant role in the prevalence of diabetes, and having a family member with diabetes increases the risk for other relatives, particularly first-degree relatives. In Saudi Arabia, consanguineous marriages are common, with a prevalence exceeding 60% in the general population and higher rates in rural areas. This practice further increases the risk of T2DM within the society (32, 33).

Thus, the higher sensitivity of HbA1c observed in our study suggests that long-term glycemic status may provide greater clinical utility in predicting CAD presence.

Smoking has been linked to dyslipidaemia and elevated blood glucose levels (34, 35). It increases total cholesterol, LDL cholesterol and triglycerides levels while reducing HDL cholesterol, making it a major risk factor for CAD (36). Evidence from animal studies suggests that exposure to cigarette smoke impairs lipid profiles and HDL functionality (37). Smoking alters lipid metabolism through multiple mechanisms: nicotine stimulates catecholamine release, leading to increased lipolysis and hepatic secretion of free fatty acids, triglycerides, and VLDL cholesterol (38). Moreover, smokers tend to consume diets higher in fat and cholesterol and lower in fibre compared with non-smokers (39). Smoking also increases plasma homocysteine levels, thereby promoting oxidative modification of LDL cholesterol, suppressing Apo A-I expression, and reducing HDL cholesterol levels (40–42).

Managing dyslipidaemia is an important component of the therapeutic management of cardiovascular diseases (43). Lipid-lowering medications target different components of dyslipidaemia and consequently affect the TG/HDL ratio. In our study, total cholesterol and serum LDL exhibited limited discriminatory value for predicting CAD in our older population compared with the TG/HDL ratio. It is possible that the statistically non-significant predictive ability of these lipid markers is due to the artificial lowering of lipid levels, which reduces their predictive power. Thus, the lack of a significant association for LDL and total cholesterol should be primarily attributed to treatment bias or confounding by indication rather than to a biological lack of importance in the elderly population.

A significant gender-based difference was observed in CAD prediction using the TG/HDL ratio, with CAD being more prevalent among males than females in our study. Consistent with our findings, a study conducted in Saudi Arabia evaluating the relationship between the TG/HDL ratio and acute coronary syndrome demonstrated a negative correlation between the TG/HDL ratio and ST-segment elevation myocardial infarction (STEMI) in men compared with women, whereas angina was more commonly observed among women (44). These gender disparities in CAD may be attributed to a combination of traditional cardiovascular risk factors, including diabetes, hypertension, obesity, and hypercholesterolemia, in addition to socioeconomic and psychosocial determinants (45). Collectively, these findings underscore the importance of incorporating sex-specific risk factors into the diagnostic evaluation and risk stratification of individuals assessed for CAD.

### 4.1 Limitations

Although this was a case–control study, the relatively small sample size represents a limitation, largely due to the recent implementation of the electronic patient record system. In addition, this was a single-center study, which limits the generalizability of the findings to the wider national population. Additionally, it would be of great value to follow up patients using a longitudinal study design to assess the prognostic value of the ratio. Moreover, the inability to establish a causal relationship between risk factors and outcomes should not be ignored. Since nearly 70% of the CAD group consists of patients with 3-vessel disease, this high disease severity may lead to an overestimation of the diagnostic performance (AUC) of the biomarkers. Therefore, the generalizability of the findings to patients with milder CAD should be carefully considered. It would be valuable to conduct a comprehensive study that stratifies the impact of various medications on the parameters by subgrouping patients according to their treatment plans.

## 5 Conclusions

Our study demonstrated that the TG/HDL ratio and total cholesterol were significantly associated with the occurrence of CAD in the Saudi population aged over 50 years; however, their sensitivity and specificity were lower than those of HDL and HbA1c as biomarkers for CAD detection. In addition, CAD was significantly more prevalent among Saudi males than females. Accordingly, the TG/HDL ratio, together with smoking status, HbA1c, and lipid markers, should be considered when assessing the prevalence and severity of CAD. Overall, our findings suggest that glycaemic control, as reflected by HbA1c, represents the most powerful biomarker for predicting CAD among adults aged ≥51 years, followed by HDL levels, whereas traditional lipid markers, such as total cholesterol and LDL, exhibit limited discriminatory value in this older population.

## Data Availability

The original contributions presented in the study are included in the article/Supplementary Material, further inquiries can be directed to the corresponding author/s.
